# Strategy actions to include students in a private hospital’s nursing teams: A qualitative study

**DOI:** 10.4102/hsag.v29i0.2533

**Published:** 2024-07-09

**Authors:** Adele Neethling, Madeleen Rheeder-Jooste, Won-Li Richardson, Lizeth Roets

**Affiliations:** 1 Faculty of Education, Life Healthcare, Johannesburg, South Africa; 2 Department of Health Studies, Faculty of Nursing, University of South Africa, Pretoria, South Africa

**Keywords:** clinical placement, experiential learning, nursing team, strategic actions, student challenges, student inclusion, team approach

## Abstract

**Background:**

Student nurse education focusses on preparing competent professionals integral to nursing teams.

**Aim:**

To develop strategic actions to include student nurses within the nursing team.

**Setting:**

A private healthcare group in South Africa.

**Methods:**

A qualitative approach in three phases was used. Thirty purposively sampled participants recorded reflective journals in phase one. Tesch’s eight steps were utilised for analysis. Phase two involved non-probability volunteer sampling of 11 participants for the nominal group technique analysis. In phase three, total population sampling recruited nine panellists for an e-Delphi technique that provided data that were open coded.

**Results:**

Phase one identified themes, including perceptions of responsibilities, support needs, recognition, conflict and communication issues. Phase two revealed five strategic actions: fair treatment, aligned work allocation, active participation, respect and acknowledgement of contributions. Phase three validated 19 strategic statements underpinning the strategic actions.

**Conclusion:**

Unit managers play a key role in fostering inclusivity, impacting student nurse retention.

**Contribution:**

Enhancing team cohesion can improve workplace wellness and patient outcomes while aiding in retention and recruitment efforts.

## Introduction

### Background

Education and training of student nurses involve more than teaching knowledge and clinical skills, as they also need to be prepared to become competent and professional nurse practitioners. Therefore, developing their social and professional skills is equally important (Pando et al. 2021:569). The educational journey of student nurses thus includes exposure to experiential learning opportunities, as stipulated by the South African Nursing Council (SANC) (SANC 2004). These opportunities are structured to ensure exposure to various clinical areas, such as medical units, surgical units and paediatric units. Exposure to patients and providing nursing care ensure that students have an opportunity not only to apply their theoretical knowledge but also to develop professional values (Feller et al. 2019:318; Hegenbarth et al. 2015:304).

Exposure to diverse clinical settings also implies that students are exposed to new nursing teams. Thus, the expectation is that they engage with and work as part of a new team while adjusting to new unit managers, professional nurses, students from other disciplines and the multi-professional team members within the clinical setting (Walker et al. 2014:104). Nursing is team based (Iddins et al. 2015:255), with no nurse working in isolation. Therefore, it is expected that when new student nurses join a team, they should be acknowledged as part of that team.

Although the roles of already qualified and professional members of the multidisciplinary team are clear, the role that the student nurse must fulfil as part of this team is neither clearly defined nor well documented (Iddins et al. 2015:259). The uncertainty about the specific role of the student in the team could affect their professional development and professional identity (Feller et al. 2019:318; Walker et al. 214:104). Students reported a lack of support from other team members during experiential learning opportunities contributing to them not feeling part of the team (Manninen et al. 2013:137). Not feeling part of the team can have an adverse influence on communication within the team, which in turn has a negative impact on efficient and effective nursing care (Aydogdu 2024:74; Baraz, Memarian & Vanaki 2015:2). Conversely, students who experienced feeling as part of a team had a sense of belonging that positively contributed to their professional growth (Walker et al. 2014:108) and quality nursing care (Dimitriadou et al. 2015:237).

### Research aim

The aim of the study was to develop a strategy to facilitate the inclusion of student nurses as part of the nursing team to enhance the learning experience.

### Research objectives

To achieve the aim, the following objective was pursued:

- Develop strategic actions based on student and unit manager input, to facilitate the inclusion of the student nurses as part of the nursing team.

### Strategy development

A ‘strategy’ can be defined as a process of selecting the most suitable action(s) to meet an objective (Cote 2020). In the context of this study, developing strategic actions that will contribute to the student nurses’ experiences of feeling as part of the nursing team. The literature describes six steps that need to be taken into consideration (Butuner et al. 2019:97; Cote 2020), and the application of these steps is illustrated in Table 1.

**TABLE 1:** Six steps of strategy development.

Step	Description	Application
Vision	Where to go	To strive towards creating an inclusive environment where student nurses are part of the nursing team
Values	How to behave	Determined by phases one and two – see Table 2 and Table 3
Focus areas	What will you be focussing on to ensure progress?	Determined by phase three – see Table 4
Objectives	What do you want to achieve?	The nursing student will feel part of the nursing team
Projects and/or actions	How will you achieve them	Determined by phase three – see discussion
Key performance indicators	How will you measure your success?	Student satisfaction

## Research methodology

### Research paradigm and design

The interpretivist approach, based on the importance of personal perspectives related to a phenomenon and the interpretation thereof (Ryan 2018:9), was applicable. The perceptions and recommendations of both student nurses and unit managers were taken into consideration for the development of the proposed strategic actions.

A generic qualitative research design was utilised, which is described by LoBiondo-Wood and Haber (2018:104) as a means to develop an understanding of a phenomenon. Qualitative research is explanatory, descriptive, inductive and contextualised in nature and was therefore appropriate within this study setting to achieve the objectives of the study.

### Research methods

This study was conducted in three phases, with different objectives to be achieved in each phase. Diverse data gathering techniques were appropriate for each phase, as illustrated in Table 2.

**TABLE 2:** Research objectives and methods.

Methods	Phase one	Phase two	Phase three
Study objective	Explore the student nurses’ perceptions of their role within the nursing team.	Describe the recommendations provided by student nurses on ways to define and clarify their role within the nursing team.	Describe the recommendations from unit managers regarding the clarification of the role of student nurses within the nursing team.
Data collection	Reflective journals	NGT	Delphi technique
Data analysis	Tesch 8 steps - 5 themes identified	Presented 5 themes - Identified 5 strategic actions	19 strategic statements supporting 5 strategic actions
Findings	Qualitative reflective journal data and literature review	Focussed on top five rated strategies (see Table 4)	Recommended strategic actions (see Table 5)

NGT, Nominal group technique.

### Study setting

The study was conducted within a health care group comprising 47 privately funded hospitals in South Africa. In phase one and two, nursing students undergoing training and placed for experiential learning in six of these hospitals based in Gauteng province were recruited. In phase three, unit managers employed in the 47 private hospitals in South Africa were recruited.

### Population and sampling

#### Population

The target population for *phase one* and *phase two* comprised 130 students enrolled in a nursing programme in a specific private nursing education institution (PNEI). The students were assigned for experiential learning opportunities to six private acute care hospitals within a specific region. The student nurses were enrolled in the bridging course (BC) programme, leading to registration as a general registered nurse (SANC 1997). Within this context, BC implies that a student is studying towards becoming a registered nurse. The target population for *phase three* was 487 unit managers, employed in 47 privately funded hospitals across South Africa. In this context, the unit manager is a nurse who obtained a diploma or degree in nursing and is employed as a manager in a nursing unit. The below mentioned criteria were applied for the selection of participants for the different phases.

#### Inclusion and exclusion criteria

Students who did not complete the four weeks in the identified units, were excluded.

*Phases one and two:* All the first- and second-year student nurses registered for the BC programme who were assigned for experiential learning in medical, surgical or paediatric units were eligible for inclusion in the study.

*Phase two:* Only students who participated in phase one were included.

*Phase three:* All 487 unit managers employed in medical, surgical or paediatric units across the 47 hospitals were invited to participate in the study.

#### Sample and sampling

A purposive sampling method, defined as the selection of participants with a particular background knowledge regarding the specific phenomenon (Grove, Gray & Sutherland 2017:317), was utilised. All students who fulfilled the inclusion criteria were invited to participate in *phase one*. In all, 30 students were eligible for inclusion in the study and received recruitment letters containing all the relevant information about the study, as well as how the ethical principles would be applied, to obtain their voluntary consent. All 30 student nurses volunteered to participate and formed the sample.

Each of the 30 students who participated in *phase one* had an equal chance of being selected and volunteered for *phase two*, thus volunteer sampling, a form of non-probability sampling was used for the nominal group discussion in phase two (Alvi 2016:28). A total of 11 students volunteered to take part in the nominal group discussion.

Total population sampling, as described by Etikan, Musa and Alkassim (2016:2), was employed to recruit participants for the e-Delphi in *phase three*. Recruitment letters were emailed to all unit managers meeting the inclusion criteria. Five unit managers, acknowledged as experts, because of their experience of working with students, volunteered as panellists in the first round of the e-Delphi process, prompted by reminder recruitment letters. The literature does not provide clear evidence of the number of panellists required when using the e-Delphi (Taylor 2020:13); thus five panellists for round one were found to be a satisfactory number (Waggoner, Carline & Durning 2016:666). After round one, all inputs and suggestions received from the five panellists were incorporated into a set of new strategic actions that were shared with the total population. Nine panellists participated in round two of the e-Delphi after which consensus was reached. It is a requirement according to the principles of the e-Delphi technique, where rounds are to continue until consensus is reached.

#### Data collection and analysis

Three phases of data collection were implemented. The findings of *phase one* informed *phase two*, which, in turn, contributed towards *phase three*.

#### Data collection

*Phase one:* Students were requested to complete a journal entry each week and reflect on instances that fostered a sense of belonging or conversely, instances that led to a feeling of exclusion. The students then had to use Gibbs’ Reflective cycle (Gibbs 1988) to describe their experience. Students completed the reflective journals over a 4-week period before submission to the researchers for analysis. Recruited participants volunteered to complete reflective journals, ensuring that there was at least one journal entry per week, as requested by the researchers.

*Phase two:* A nominal group technique (NGT) suitable for qualitative data collection, which balances the influences of all participants by allowing each participant an equal voice to share ideas until consensus is reached, was used (Van de Ven & Delbecq 1972). The NGT was facilitated by an expert external facilitator with experience and knowledge of NGT data collection and analysis. A venue agreed upon by all participants was selected at one of the PNEI. Eleven participants took part, which is an appropriate number of participants for NGT (Waggoner et al. 2016:664). The NGT was conducted in person over two sessions. Session one entailed a PowerPoint presentation to share the findings from *phase one*. The question ‘What would make you feel part of the nursing team?’ was posed to allow a focussed discussion for session two.

The principles of data gathering, according to Foth et al. (2016:11) were followed. A classic four-step NGT process was followed, namely: (1) round-robin method of data generation (generation of ideas), (2) identification of themes, (3) clarification of themes and (4) voting and ranking the most important to the least important (McMillan et al. 2016:655). This process took 2 h to complete.

*Phase three:* The Delphi technique was utilised in this phase and is a systematic consensus method of obtaining data from a panel of experts, who agree on a particular issue or item of research (Humphrey-Murto et al. 2017:14). This method includes the use of controlled feedback between the researchers and a group of experts or panellists to obtain a group opinion (Trevelyan & Robinson 2015:423). The e-Delphi technique is a means of conducting the Delphi technique electronically with a computer and/or with online analysis software (Green 2014:1).

The findings of *phase one*, a literature review and focussed top five rated strategies from the nominal group analysis (phase 2), contributed to the development of the strategic actions to be validated with the e-Delphi technique in *phase three*. Based on the identified strategies, the authors imbedded a validation tool to allow panellists from the e-Delphi to provide inputs and comments to improve suggested strategic actions.

The strategic actions and validation tool were loaded on SurveyMonkey™. The recruitment letter shared with panellists gave them access to the validation tool. Two rounds of e-Delphi were needed for consensus. Consensus was reached when 80% of panellists agreed to a specific item or strategy (Waggoner et al. 2016:667).

#### Data analysis

**FIGURE 1:**
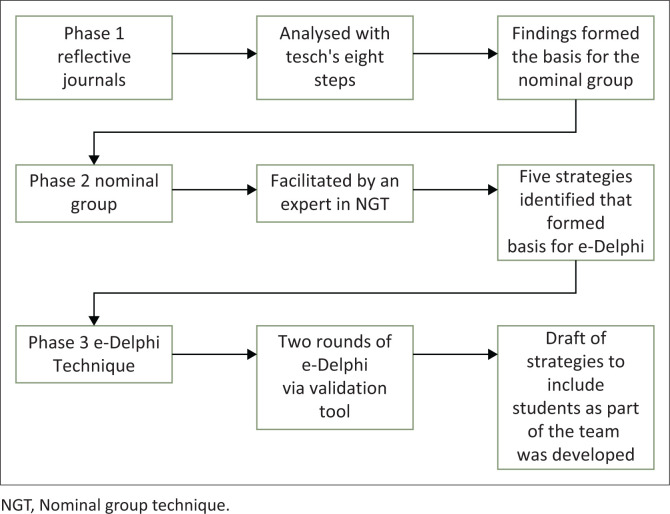
Data analysis process flow.

Figure 1 illustrates the data analysis process that was followed from phase one through to phase three.

Data from *phase one’s* reflective journals were analysed using Tesch’s eight steps for qualitative data analysis (Creswell & Creswell 2018:271) (Table 2 and Figure 1). These findings were presented to participants in *phase two* to provide a background for the participants who took part in the NGT. The justification for providing a background presentation to guide the NGT stemmed from the fact that the students, despite their involvement in phase 1, were unaware of the findings from other participants. This presentation served to offer participants a comprehensive overview of the collected perspectives and experiences within the group. By doing so, it facilitated their ability to concentrate on essential aspects when formulating the strategy. The nominal group analysis, displayed in Table 3, revealed five top rated strategies. These strategies were used to draft strategic actions to facilitate the inclusion of student nurses in the nursing team for *phase three*.

**TABLE 3:** Themes, categories and sub categories identified during phase one.

Theme	Sub themes	Code	Categories
Too many responsibilities	Routine tasks	P4, Med	‘I was allocated to do emergency trolley and Counting of ward stock’.
		P6, Sur	‘The unit was very busy. I was responsible for admission, post-operative care for patients’.
		P14, Med	‘Bad experience working as Workforce limiting the time to learn and meet some of my objectives’.
	Workload	P6, Sur	‘There was a shortage of staff I was responsible for nursing surgical patients as surgical ward was full’.
		P7, Paed	‘I felt I was not coping I was even telling my colleague allocated the side opposite to mine that I think I will be knocking off at 9 o’clock today’.
		P17, Sur	‘I feel that the registered nurse in the ward sometimes put all the responsibilities to students rather than working with them and teaching them’.
	Managerial tasks	P3, Sur	‘I was allocated with new staff member who were not familiar with [*a private*] hospital [*group*] document and the way we do things in the hospital. They needed more orientation but it was time consuming’.
		P9, Med	‘The good experience that I was exposed to be the shift leader and carrying out duties of a registered nurse delegating as well’.
		P22, Med	‘I was happy to get the opportunity to attend a meeting with the matron and the unit managers’.
Need of support	Supernumerary	P17, Sur	‘Students are unable to learn some of the things that are to learn while in the wards because their duties are to do vital signs and admissions’.
		P27, Med	‘Matron called the unit manager and asked if she knew that I just called Dr E’. (The student stated that students are not allowed to phone the doctor)
		P7, Paed	‘Student’s we are Flex to go and work in other words and ending up not learning anything’.
		P25, Sur	‘When it is busy in the unit is my position as a student disregarded, and is supervision also disregarded?’
	Need for orientation	P3, Sur	‘2nd week in the unit. Unit manager is back to work from sick leave but still no orientation to the ward ask if I did receive orientation but still never orientated though’.
		P4, Med	‘Poor orientation and poor communication skills can really make one’s experience very poor’.
		P3, Sur	‘On duty on this day and the shift leader on duty welcomed me and introduced me to the new unit manager’.
	Mentoring	P3, Sur	‘The first day I worked closely with the shift leader which she guided me on everything’.
		P7, Paed	‘Work within my scope of practice and Seek assistance from my Seniors where I needed it’.
		P6, Sur	‘It was working under direct supervision of the unit manager and she was also admitting and administering medication with me’.
		P25, Sur	‘I find again that there is not enough supervision of a student but we are expected to carry a lot of responsibility which does not tie together’.
	Lack of confidence	P11, Sur	‘Not even one person who came to see if I was coping or not. Or if I needed assistance’.
		P18, Paed	‘There is no team work and that lots of nurses go through lots of things but we are scared to report them’.
	Unrealistic expectations	P2, Sur	‘He asked why I’m not answering the bell but he saw that I was busy the patient’.
		P10, Paed	‘She Told me that she gave me results to give to the doctor and I never did and now she is in trouble because of me’.
		P28, Sur	‘He was Very upset it was screaming and shouting. I was not responsible to fill out his forms’.
		P11, Sur	‘It was bad because they made me to deceive [*receive*] the patient alone while they sat at the duty station not even one person who came to see if I was coping’.
		P10, Paed	‘He was comparing us to another Hospital where he is working’.
Acknowledge the role of the student	Respect	P7, Paed	‘Patient was called to theatre and there was no X-rays done the doctor came back to the word extremely Furious it started shouting at every staff member in the ward’.
		P15, Paed	‘The great amount of respect for me as a person and within the profession is unbelievable’.
		P3, Sur	‘Compliments from the patients and the staff and unit manager and appreciation to the staff members boosted the morale of the nursing staff’.
		P19, Med	‘The sister in charge shouted the student as she enters the unit in front of everyone’.
Conflict	Blaming students	P2, Sur	‘The progress report was not written I was on teatime. I felt that there was no teamwork because the sister told me to go’.
		P4, Sur	‘Confessed after I was shouted at for not being competent in what I’m doing given the opportunity for my side of the story’.
		P20, Med	‘To my surprise she gave me an attitude that I was the one that received the patient from ICU I must carry the orders and it was already 4 o’clock’.
	Conflict with other team members	P6, Sur	‘The gynaecologist called to give orders regarding medication and I asked him to hold, calling the registered nurse to take the orders and that made him unhappy’.
		P7, Paed	‘The doctor came and started shouting and insisted that the head must be shaved’.
Lack of communication	Communication between professionals and shifts	P11, Sur	‘She only asked me when I was about to knock off why was the patient admitted so she can hand over to the night staff. The following morning the medication was not given’.
		P26, Med	‘The enrolled nurse that was handing over did not hand over property [*properly*]’.
		P18, Paed	‘This situation would have been resolved sooner if the staff in the unit has communication or a handover’.
		P5, Med	‘Importance of communication amongst the staff, especially after doctors’ rounds’.
	On-duty schedule	P8, Paed	‘I politely discussed or suggested my hours were more she said ‘I have been working with students for long do you mean that I don’t know my work?’
		P11, Sur	‘She told me the unit manager was not available but the off duties are done by the ward clerk she said she was in the kitchen having lunch’.
		P7, Paed	‘This week I did not feel as part of the team because I did not have off duties’.

P, participant; Sur, surgical unit; Med, medical unit; Paed, paediatric unit.

In *phase three*, participant responses received from SurveyMonkey™ software were analysed based on the five participants who responded. Consensus was determined if at least 80% of the participants agreed with the presented strategic actions. Strategic action statements that did not obtain 80% consensus were adapted and presented to the whole population again for a second round of consensus for all strategic action statements. Nine participants responded to the second round recruitment letter and e-Delphi. After round two, an 80% consensus was achieved, and strategic actions for student inclusion in nursing teams were accepted.

#### Ethical considerations

Ethical approval to conduct this study was obtained from the National Health Research Ethics Committee registration: REC 251015-048. The ethical clearance number is 08272021/1, and informed written consent from both student participants and unit managers panellists were obtained before data gathering commenced. All participants received information letters through gatekeepers. The information letters included detailed information about the study, the study purpose, participant roles and responsibilities, as well as how the researchers would apply ethical principles to protect the participants, panellists and the private health care group. The ethical principles described by Brink, Van der Walt and Van Rensburg (2018:28) were applicable. The relevant principles of respect for individuals, autonomy, beneficence, justice, privacy and confidentiality were adhered to.

## Results

### Phase one: Student narratives (reflective journals)

Students’ narratives were analysed, as described under the previous sections on data collection and data analysis. Five themes and 18 subthemes were identified from the data. To portray how the subthemes were grouped from the categories, the direct quotations are displayed in Table 3.

### Phase two: Nominal group findings

Eleven participants volunteered to take part in the nominal group, facilitated by an NGT expert, as described in the section on data collection and analysis. Several rounds of data generation continued until no new ideas were shared pertaining to the question, ‘What would make you feel part of the nursing team?’. All the ideas were grouped into themes and, as a team, all the participants contributed to the thematic analysis until they agreed on the identified themes. The participants then voted for the themes and rated them from the highest priority to the lowest (see Table 4). These themes formed the strategic actions.

**TABLE 4:** Strategic actions identified during nominal session.

Themes	Total number scored during the voting session
1. Fair and equal treatment	27
2. Work allocation to meet student’s objectives	24
3. Active participation in all learning opportunities	22
4. Respect students as individuals	17
5. Acknowledge students’ contributions to nursing care	13

#### Identification of strategy statements

The research findings shown in Table 4, as well as appropriate literature, formed the basis for the suggested actions underpinning the strategy. These suggested actions, embedded in the validation tool, were shared with the e-Delphi panellists. The validation of the actions allowed all panellists an equal voice pertaining to what actions could be suggested to facilitate a positive experience for student nurses to feel part of the nursing team.

### Phase three: e-Delphi validation findings

The validation tool allowed all panellists either to agree or disagree with a specific strategy action, as well as gave them an opportunity to provide narrative data to improve these actions, change them or provide additional information. Two rounds of e-Delphi were needed to achieve 80% consensus. Findings from round one are displayed in Table 5.

**TABLE 5:** Panellists’ responses during round one (*N* = 5).

Priority themes from phase 2		Strategy actions (S)		Agree		Neither agree nor disagree		Disagree		Consensus
				*n*	*f*(%)	*n*	*f*(%)	*n*	*f*(%)	
2	Work allocation to meet student’s objectives	S1	Complete the students-on-duty schedule before the student starts working in the unit.	5	100	0	0	0	0	Yes
2	Work allocation to meet student’s objectives	S2	Allocate structured clinical days on the on-duty schedule	5	100	0	0	0	0	Yes
4	Respect students as individuals	S3	Introduce the students to the nursing team on their first day in the unit, using the students’ rank and first name.	2	40	2	40	1	20	No
2	Work allocation to meet student’s objectives	S4	Have a meeting with the student and student mentor on the first day that the student joins the unit to determine the student’s objectives.	5	100	0	0	0	0	Yes
4	Respect students as individuals	S5	Have a meeting with the student before they join the unit to confirm their clinical days.	3	60	0	0	2	40	No
3	Active participation in all learning opportunities	S6	Have a meeting with the student and student mentor on the first day that the student joins the unit to communicate expectations from the Unit Manager or shift leader.	4	80	0	0	1	20	Yes
2	Work allocation to meet student’s objectives	S7	Have a meeting with the student and student mentor on the student’s first day of joining the unit to determine student’s expectations.	4	80	0	0	1	20	Yes
4	Respect students as individuals	S8	Assign a dedicated student mentor to the students per shift.	4	80	1	20	0	0	Yes
2	Work allocation to meet student’s objectives	S9	Registered nurse to consider student objectives when delegating	4	80	0	0	1	20	Yes
4	Respect students as individuals	S10	Communicate student objectives daily.	4	80	1	20	0	0	Yes
3	Active participation in all learning opportunities	S11	Ensure students are paired with a buddy who will assist them to practise their skills to reach their clinical objectives.	5	100	0	0	0	0	Yes
2	Work allocation to meet student’s objectives	S12	Openly acknowledge student contributions to patient care by posting positive comment cards on the notice board.	4	80	1	20	0	0	Yes
4	Respect students as individuals	S13	Openly acknowledge student contributions to patient care by being open to students’ suggestions regarding possible patient care improvement (i.e., shift ideas).	5	100	0	0	0	0	Yes
3	Active participation in all learning opportunities	S14	Openly acknowledge student contributions to patient care by including students in reward and recognition nominations if they have excelled in the unit.	5	100	0	0	0	0	Yes
2	Work allocation to meet student’s objectives	S15	Communicate student objectives to the rest of the nursing team through visible communication strategies.	5	100	0	0	0	0	Yes
4	Respect students as individuals	S16	Identify individual staff who are involved in student bullying.	4	80	1	20	0	0	Yes
3	Active participation in all learning opportunities	S17	Manage individual staff who are involved in student bullying.	5	100	0	0	0	0	Yes
2	Work allocation to meet student’s objectives	S18	Ensure Unit Manager and/or student mentor has a feedback meeting at the end of the student’s placement to ensure that clinical objectives have been met.	4	80	1	20	0	0	Yes

After receiving feedback from the panellists, a second round of data collection was needed, as consensus was not reached on all statements. The validation tool was adapted based on the suggestions for improvement received from the panellists. A recruitment letter providing access to the changed strategic action statements was sent to the total population for input during round two as displayed in Table 6. Suggestions for inclusion of strategic actions pertaining to setting clinical objectives for the unit, opportunities and planning on-duties, accordingly, were included in the second round (S19). Nine panellists responded to the recruitment letter for round 2.

**TABLE 6:** Unit managers’ responses during round two (*N* = 9).

Strategy actions (S)	Agree		Neither agree nor disagree		Disagree		Consensus obtained
	*n*	*f*(%)	*n*	*f*(%)	*n*	*f*(%)	
S1 and S2. Consensus reached in round 1.	-	-	-	-	-	-	-
S3. Introduce the students to the nursing team on their first day in the unit, using the students’ rank, name and surname.	9	100	0	0	0	0	Yes
S4. Consensus reached in round 1.	-	-	-	-	-	-	-
S5. Have a meeting with the student before they join the unit to confirm their clinical days.	9	100	0	0	0	0	Yes
S6–S18. Consensus reached in round 1.	-	-	-	-	-	-	-
S19. Identify unit-specific learning opportunities and plan duties accordingly. (Action added from round 1)	8	89	0	0	1	11	Yes

### Trustworthiness

Lincoln and Guba’s (1986:172–173) methods of credibility, dependability, confirmability and transferability were used to ensure trustworthiness. Credibility was demonstrated by fostering an open communication channel with the participants, allowing them to ask questions for clarification, thereby enhancing the trustworthiness of the study. Additionally, trust was cultivated through the conscientious adherence to appointments and scheduled NGT sessions. Moreover, trustworthiness was ensured by member checking and collaborative meetings with all researchers involved. These discussions led to a consensus on the study’s overarching themes and subthemes, mitigating errors and ensuring that the findings were firmly representative in participants’ quotations. Dependability was upheld through the selection of an appropriate research paradigm and methodology that were considered most suited for the study’s objectives. This ensured the consistency and reliability of the research. To enhance confirmability, all collected data sets were collaboratively checked and re-checked by co-researchers to ensure rigorous and accurate coding processes. Transferability was ensured by a thorough description of the research methodology.

## Discussion

Student nurses should be incorporated as members of the nursing team to assist them in transitioning from having only theoretical knowledge to the application thereof in nursing practice. At the same time, they should be introduced to the professional team of nurses of which they will become an integral part after completion of their studies. Being part of the nursing team as a student will enhance the students’ professional growth (Church, White & Cosme 2019:174). It is the responsibility of a unit manager, sometimes referred to as a nurse leader, to support student nurses and introduce them to the nursing team (Human & Mogotlane 2017:18).

Student nurses require diverse experiential learning opportunities, for which they are allocated to different clinical settings as required by the SANC. During their pursuit of clinical competencies, they are also expected to grow into professionals by acquiring professional behaviour (SANC 1997). In order to ensure that student nurses feel part of the nursing team, the panellists reached consensus on specific strategic actions as described.

### Fair and equal treatment

Being part of the nursing team, under the supervision of unit managers and other professional nurses, provides students with the ideal opportunity for professional growth and development. Participant stated:

‘It was working under direct supervision of the unit manager and she was also admitting and administering medication with me’. (Participant 6, Surgical unit)

For students to feel part of the team, they must feel valued and not feel abused by the staff in the unit (Jack et al. 2017). Students who experience hostility, rejection and unfair treatment from the team become despondent and withdrawn, which leads to their low participation in the daily activities and hindering the development they need to become a professional registered nurse (Mohamed 2019:500). Bullying of students still occurs, despite being criticised nationally (Birks et al. 2018). Another participant expressed their frustration as follows:

‘Confessed after I was shouted at for not being competent in what I’m doing given the opportunity for my side of the story’. (Participant 4, Surgical unit)

Unit managers are in the ideal position to make students feel part of the team by displaying an inclusive attitude. They, among all other responsibilities, must be available and approachable, treating all those joining the unit with respect and being receptive to new ideas (Nouri et al. 2019). Students who enjoy their time in a specific unit during their student years and who feel part of the team are more likely to return to that unit after obtaining an official qualification (Rodríguez-García et al. 2021). Regarding strategic actions S16 and S17, the unit managers agreed that students should be treated fairly.

### Work allocation to meet students’ study objectives

Unit managers should meet with the student at the commencement of their first shift to discuss the student’s objectives received from the PNEI. Church et al. (2019) describe the benefits of following structured plans when assisting students in the clinical area. Strategic action statements five and nine (S5 and S9) refer to the allocation of student objectives at the start of the shift. When staff members are not aware of the student’s outcomes, their learning suffers and they often end up doing routine tasks, as expressed by one participant:

‘Students are unable to learn some of the things that are to learn while in the wards because their duties are to do vital signs and admissions’. (Participant 17, Surgical unit)

### Active participation in all learning opportunities

When student nurses are committed to learning, they display openness, willingness and active participation in gaining new knowledge. However, although student nurses may be active in their participation in learning, it is equally important for mentors to be actively involved in the students’ learning process (Tsimane & Downing 2020:94). As one participant asserted:

‘The first day I worked closely with the shift leader which she guided me on everything’. (Participant 3, Surgical unit)

This indicated that a mentor was actively involved in her learning. This statement also ties in with the strategic action statements suggesting that students be buddied with a mentor to discuss their expectations and objectives on their first day of clinical placement in the unit. Furthermore, mutual participation and meaningful interaction between student nurses and mentors within the nursing team produce an increase in knowledge and help in developing critical thinking skills (Tsimane & Downing 2020:95).

### Respect students as individuals

The literature has highlighted that student nurses value relationships with mentors in the nursing team that are constructive and mutually respectful. As a result, students should be invited to share their thoughts and ideas, while the other team members should show appreciation for them as valuable and equal team members (Dale, Leland & Dale 2013:5). Furthermore, student nurses value being respected as both individuals and professionals (Dale et al. 2013:6). A participant asserted:

‘The great amount of respect for me as a person and within the profession is unbelievable’. (Participant 15, Pediatric unit)

When student nurses feel welcomed and are met with friendliness when commencing their clinical placements, they feel a sense of belonging and acceptance in the unit (Dale et al. 2013:6). This view is supported by the strategic action statements relating to introducing the students to the nursing team on their first day in the unit, as well as arranging meetings with the students and their mentors to discuss the student’s expectations and communicating student objectives daily to the nursing team.

### Acknowledge students’ contributions to nursing care

As student nurses form part of the nursing team in the clinical unit where they are placed (Iddins et al. 2015:261), they not only want to reach their learning objectives but also receive appreciation for the role they play in patient care (Singer, Sapp & Baker 2022:103). As one participant (Participant 25, Surgical unit) indicated that they carry a lot of responsibility. This signifies that there is a need for students to be acknowledged for their contribution. The effect that a ‘Thank you’ can have on morale is immense. One participant wrote in the reflective journal:

‘Compliments from the patients and the staff and unit manager and appreciation to the staff members boosted the morale of the nursing staff’. (Participant 3, Surgical unit)

This view is supported by Iddins et al. (2015:261), who found that regular thanking of individuals and affirmation of their contributions are important for reaching team goals. The inclusion of focussed strategy actions to acknowledge students as part of the team will contribute to their sense of belonging in the team.

### Recommendations

Unit managers must adopt or adapt the recommended strategic actions in clinical practice to ensure that student nurses feel as part of the team, thus promoting their professional growth. A further recommendation is to validate this study in other health care contexts to determine the viability of the recommended strategic actions. In addition, training and induction programmes for unit managers should emphasise the importance of creating an inclusive environment in clinical practice.

## Conclusion

The study findings revealed that student nurses want to feel part of the nursing team but do not always experience that. Nevertheless, it is important for the nursing profession that all student nurses be developed socially and professionally. The avenue for reaching this objective is through experiential learning, whereby student nurses experience first-hand how unit managers, as professional practitioners, act within the nursing team. Unit managers therefore have a responsibility not only to include the students in the team but also to acknowledge their contributions as team members. Moreover, unit managers should defend and protect the students when they are disrespected by other members of the multi-professional team.
